# Sijunzi Decoction attenuates 2, 4, 6-trinitrobenzene sulfonic acid (TNBS)-induced colitis in rats and ameliorates TNBS-induced claudin-2 damage via NF-κB pathway in Caco2 cells

**DOI:** 10.1186/s12906-016-1549-3

**Published:** 2017-01-10

**Authors:** Yue Lu, HanJie Lin, JinWei Zhang, JianAn Wei, Jing Sun, Ling Han

**Affiliations:** 1 The Second Clinical College, GuangzhouUniversity of Chinese Medicine, Guangzhou, 510000 China; 2 Guangdong Provincial Hospital of Chinese Medicine, Guangzhou, 510000 China

**Keywords:** Claudin-2, Inflammatory bowel disease, NF-κB pathway, SijunziDecoction, Tight junction

## Abstract

**Background:**

SijunziDecoction (SJZD) is a traditional Chinese medicine prescription used to treat the diseases of gastrointestinal tract since ancient times. The objective of this study was to investigate the protective effects of SJZD on TNBS-induced colitis in rats and TNBS-damaged Caco2 cells.

**Methods:**

The rat colitis model was induced by 2, 4, 6-trinitrobenzene sulfonic acid (TNBS). SJZD (2.8 5.6, 11.2 g/kg) or salazosulfapyridine (SASP) (0.4 g/kg) was administrated orally in rats for 7 days. DAI, pathological scores and the expression of claudin-2 were evaluated. Then we explored the effect and mechanism of SijunziDecoction Serum (SJZDS) onTNBS-damaged Caco2 cells to figure out intestinal barrier protective effect and mechanism of SJZD.

**Results:**

SJZD significantly ameliorated the severity of TNBS-induced colitis and downregulated the level of claudin-2 in colonic tissues. SJZDS promoted proliferation and inhibited apoptosis ofTNBS-damaged Caco2 cells. In Caco2 cell monolayers, we provided mechanistic evidence that SJZDS-induced increased TEER and decreased permeability after TNBS damage, which were mediated through claudin-2 and NF-κB pathway, including the upregulation of claudin-2, decreased activity of NF-κB p65, reduced level of NF-κB p65 and MLCK.

**Conclusions:**

Our results indicated that SJZD possesses protective effect of intestinal barrier towards TNBS-induced colitis in rats and TNBS-damaged Caco2 cells in vitro. SJZDis a potential protective agent of intestinal barrier that deserves further investigation.

## Background

Inflammatory bowel diseases (IBDs) are chronic recurrent inflammatory diseases of the gastrointestinal tract, mainly including Crohn’s disease (CD) and ulcerative colitis (UC) [1, 2]. The development of IBDs is linked to characteristic changes in the colon, such as epithelial cell necrosis and ulceration, local infiltration by immune cells, including a significant number of neutrophils and T cells [3]. The ongoing activation of the mucosal immune system are considered to be the major pathological agents of IBDs [4].

The epithelium of the mucosa plays an essential role in maintaining balance of intestinal ecosystem [5]. The tight junction complexes play a decisive role in the maintenance of barrier integrity [6]. Tightjunctions are multi-protein complexes, which composed of transmembrane proteins, peripheral membrane (scaffolding) proteins and regulatory molecules. The claudin protein family is the most important transmembrane proteins, which can impact the tight junction permeability. The transmembrane proteins also include occludin, which interacts directly with claudins and actin. Zonula occludens 1 (ZO1) and ZO2 are peripheral membrane proteins which play a crucial role on tight junction assembly and maintenance [7].

Currently, conventional drugs used for treatment of IBD are including immunosuppressants, corticosteroids, disease improving agents and biological agents, such as TNF antibodies. However, most of these medicines only temporarily alleviate the symptoms, and are associated with mild to serious side effects, leading to their limited clinical applications [8]. Thus traditional Chinese Medicine Formulas gaining more and more attention in the treatment of IBD, due to its effective and safe [9]. Some clinical and experimental studies have shown that Chinese formulas for treatment of IBD were effective, and most of their mechanisms were related with anti-inflammatory, anti-oxidant and restore the function of intestinal barrier [10–12].

Sijunzi Decoction (SJZD),also known as Four Gentleman Decoction, is a classical prescription for curing spleen deficiency in traditional Chinese medicine, consisting of *Ginseng Radix et Rhizoma, or Codonopsispilosula, Atractylodes Macrocephalae Rhizoma, Poria, and Glycyrrhizae Radix et Rhizoma Praeparatecum Melle.* Modern pharmacological experiments have proved that saponin, flavonoid, and polysaccharide are the most active ingredients in SJZD [13]. SJZD has been used for years in China to regulate the gastrointestinal function and enhance the immunity [14]. However, molecular mechanisms by which SJZD suppressed inflammation bowel disease were unclear. In the present study, we aimed to investigate the therapeutic efficacy of SJZD against IBD. Also, we analyzed the expression of tight junction related protein to investigate the mucosal barrier protective mechanism of SJZD in vitro.

## Methods

### Reagents and chemicals

DMEM medium, fetal bovine serum (FBS) and NEAA were purchased from GIBCOLaboratories (Grand Island, NY, USA). 2, 4, 6-trinitrobenzene sulfonic acid (TNBS), MTT were purchased from Sigma-Aldrich (St. Louis, Mo, USA). Salazosulfapyridine (SASP) was purchased from Tongda Pharmaceutical Company Ltd. (Datong, Shanxi, China). The anti-claudin 2 antibody, anti-myosin light chain kinase antibody and NF-κB p50/p65 transcription factor assay kit were all obtained from Abcam (Cambridge, MA, USA). Trizol and cDNA synthesis kit were obtained from Invitrogen (Carlsbad, CA, USA). The RT-PCR primers were synthesized by Invitrogen (Shanghai, China).

### Plant materials

Sijunzi Decoction (SJZD) was composed of *Ginseng Radix et Rhizoma, Atractylodes Macrocephalae Rhizoma, Poria, and Glycyrrhizae Radix et Rhizoma Praeparate cum Melle*, and these four drugs were in same ratio as described in Pharmacopoeia. Dried herbs were purchased from Kangmei Pharmaceutical Company Ltd. (Guangzhou, Guangdong, China).

### Preparation of Sijunzi Decoction (SJZD)

The preparation of concentrated water decoction of SJZD was as follows: The herbs (composed of *Ginseng Radix et Rhizoma, Atractylodes Macrocephalae Rhizoma, Poria, and Glycyrrhizae Radix et Rhizoma Praeparate cum Melle*, the ratio of four herbs was 3: 3: 3: 2 and the total quantity was 216 g) were placed in a container, and soaked in cold water of about 7 times the amount of herbs for 2 h; the herbs were boiled for 30 min and then filtered. Next, the herbal drugs were added with water of about 5 times the amount of the drugs, then decocted and boiled for 30 min and filtered again. At last, the two filtrates were mixed together. Three drug concentrations (1.12 0.56 and 0.28 g/ml) were prepared and stored at 4 °C.

### Preparation of Sijunzi Decoction-serum (SJZDS)

Twenty male SD rats (6–8 weeks old, 180–200 g) were purchased from the Experimental Animal Center of Guangdong Provincial Hospital of Chinese Medicine. Rats were divided randomly into two groups and were given SJZD or equivalent volume of saline. Rats were administered orally with SJZD at 0.5 g/kg twice a day for 4 days. Then, 1.5 h after the last administration, blood of the rats was collected from the abdominal aorta under ether anesthesia. Blood from the same group was mixed and centrifuged to obtain serum. The serum was heat-inactived at 56 °C and sterilized by membrane filtration before use. The serum of rats administered with vehicle alone was also prepared as control.

### Animals

Female Wistar rats (6–8 weeks old, 180–200 g) were obtained from the Experimental Animal Center of Guangdong Province (Guangzhou, China). All rats were fed on a standard diet, had free access to water and were housed under standard laboratory conditions. All experiments were approved by the Animal Welfare and Ethics Branch of the Biomedical Ethics Committee of Guangzhou University of Chinese Medicine.

### Induction of colitis

Colitis was induced using the TNBS, as described previously. The rats that had been fasted for 24 h with free access to water were anesthetized with 10% chloral hydrate, and a polyethylene catheter (2 mm in outer diameter) was inserted rectally (6–8 cm from the anus). Then, 100 mg/kg TNBS dissolved in 0.25 ml of 50% ethanol was administered through the catheter. After administration of TNBS, rats were held in headfirst position for 3 min to prevent the solution leaking out. Then, the animals were placed in separate cages with free access to food and water. Control rats received 0.9% saline. Stool consistency and occult blood were recorded daily. Rats were sacrificed after 7 days treatment. The colonic tissues were quickly removed and rinsed with ice-cold PBS.

### SJZD administration

After a 1-week adaptive period, rats were randomly dividedinto four groups (*n* = 8) as follows. (1) The control group and TNBS group received 2 ml saline via the rectum; (2) The TNBS + low dose of SJZD group received SJZD at concentration of 2.8 g/kg;(3) The TNBS + medium dose of SJZD group received SJZD at concentration of 5.6 g/kg; (4) The TNBS + high dose of SJZD group received SJZD at concentration of 11.2 g/kg;(5) The TNBS + SASP group received SASP at concentration of 0.4 g/kg; Drug administration to animals began 24 h after induction of colitis and was treated for 7 days.

### Histological assessment of colitis

Formalin-fixed colon sections were embedded in paraffin and sectioned (7 μm), then stained by hematoxylin and eosin (H&E). Histopathologic severity was scored using three different parameters as previously described: severity of inflammation (0, none; 1, mild; 2, moderate; 3, severe); extent of inflammation (0, none; 1, mucosa; 2, mucosa and submucosa; 3, transmural);crypt damage (0, none; 1, basal one-third damaged; 2, basal two-thirds damaged; 3, crypt lost but surface epithelium present; 4, crypt and surface epithelium lost). All parameters were scored by two independent observers in a blinded manner.

### Immunochemistry

The sections were dewaxed by heating at 55 °C for 30 min, washed twice for 15 min, rehydrated in ethanol for 15 min, proceeded in water bath at 95 °C for 5 min for antigen unmasking, and treated with 3% hydrogen peroxide for 30 min. The sections were then incubated at 4 °C overnight with anti- claudin-2 antibody diluted 1:500. Next, the sections were washed with PBS and incubated secondary antibody at 37 °C for 30 min. 3’3-diaminobenzidine tetrachloride was added to observe the positive expression. Negative control sections were incubated with PBS instead of primary antibody. The expression of claudin-2 was quantified by image-pro plus software.

### Cell culture

The human colon adenocarcinoma cell line Caco2, purchased from the Cell Culture Unit of Shanghai Scicence Academy (Shanghai, China), Cells were grown in DMEM supplemented with 10% FBS and 1% NEAA in a humidified 5% CO2 atmosphere at 37°C.

### TNBS damage model of cells

For TNBS damage model of cells, the Caco2 cells were treated with 200 μg/ml TNBS for 24 h. The Caco2 cells incubated with DMEM media were used as control.

### Cell viability assay

Cell viability was determined by the MTT assay. Cells were plated at a density of 5000 per well in 96-well plates in DMEM + 10% FBS + 1% NEAA and then treated with 200 μg/ml TNBS. After exposure to TNBS for 24 h, SJZDS or control serum (cs) was added for 24 h, then10 μl MTT solution was added for 4 h. The cells were lysed with 0.04 N HCL in isopropyl alcohol, and the absorbance was read at 570 nm. Cell viability was calculated as follows: cell viability (%) = absorbance of test group/absorbance of controlled cell group × 100%.

### Hoechst 33342 staining

For analysis of chromatin condensation or nuclear fragmentation, SJZDS-treated cells after TNBS damage were harvested and fixed in 3% paraformaldehyde (PFA) for 20 min, incubated in 0.1% Triton-X100-PBS for 30 min, then stained in 0.25 μg/ml Hoechst 33342 for 15 min. The cells were observed using the IX70 fluorescence microscope (Olympus, Japan).

### Electrical resistance measurements

Transepithelial electrical resistance (TEER) of Caco2 cells was monitored using an EVOM TEER meter (Millipore). TEER increased until day 7 when a steady state of higher than 200 Ω cm^2^ was reached, indicating the formation of complete tight junction, an intact monolayer and maximized integrity of barrier function [15]. TNBS stimulation of Caco2 cells lasted for 24 h until addition of SJZDS. After removal of TNBS, the Caco2 cell monolayers was washed with PBS, numerical readings of TEER were recorded at 24 h after the co-incubation of Caco2 cells and SJZDS.

### Permeability study

All transport studies were conducted at 37 °C, in a medium of DMEM + 10% FBS + 1%NEAA. Prior to the transport study, cell monolayers were washed with PBS, phenolsulfonphthalein at final concentrations of 20 mg/L in distilled water was added to the apical compartment. The basolateral compartment contained only water. After 4 h, 150 μl of samples were removed from the basolateral compartment into 2 ml EP tubes which possess 1.5 ml NaOH (20 μmol/ml) for neutralization. The absorbance at 570 nm was measured.

### Real time-PCR analysis

The SJZDS-treated Caco2 cells were harvested at 24 h after the addition of TNBS or vehicle to analyze the expression of ZO-1, ZO-2, ZO-3, claudin-1, claudin-2, claudin-3, claudin-4, occludin, JAM, E-cadherin and GAPDH mRNAs. Total RNA was isolated using Trizol reagent. RNA purity and concentration were assayed with a NanoDrop 2000 devise (Thermo Scientific, Wilmington, DE, U.S.A.). The mRNAs were then reverse transcribed directly into cDNA using a RT-PCR kit according to the manufacturer’s instructions. PCR amplification conditions were as follows: initial denaturation at 95 °C for 15 s followed by 35 cycles of denaturation at 95 °C for 5 s and annealing at 61 °C for 15 s. The sequences of the respective sense and antisense primers were as follows (from 5’to 3’): CAACATACAGTGACGCTTCACA and CACTATTGACGTTTCCCCACTC for ZO-1; ATGGAAGAGCTGATATGGGAACA and TGCTGAACTGCAAACGAATGAA for ZO-2; GCTTTGGCATTGCGATCTCTG and GATGTGGTCGCCTGTCTGTAG for ZO-3; AGGAATTAACTGCATACGTTTTGG and TAGCCACAGAAAGCATCGGG for claudin-1; GCCTCTGGATGGAATGTGCC and GCTACCGCCACTCTGTCTTTG for claudin-2; AACACCATTATCCGGGACTTCT and GCGGAGTAGACGACCTTGG for claudin-3; TGGGGCTACAGGTAATGGG and GGTCTGCGAGGTGACAATGTT for claudin-4; ACAAGCGGTTTTATCCAGAGTC and GTCATCCACAGGCGAAGTTAAT for occludin; TGTTTCAGTTCTGTGTCATGGT and TGCAGACAAGGTGTTTTCCAG for JAM; CGAGAGCTACACGTTCACGG and GGGTGTCGAGGGAAAAATAGG for E-cadherin; GGAGCGAGATCCCTCCAAAAT and GGCTGTTGTCATACTTCTCATGG for GAPDH. Relative mRNA quantities were determined by using the 2ΔΔCt method with data normalized to the GAPDH housekeeping gene.

### Immunofluorescence

Caco2 cells were seeded in the wells of 6-well plates and cultured overnight. TNBS damaged cells were treated with SJZDS (15%) for 0, 24, 36, 48 h. After the treatment of SJZDS, Caco2 cells were fixed in 3% paraformaldehyde (PFA) for 30 min, incubated overnight at 4 °C with anti-claudin 2 antibody, followed by incubation for 1 h with anti-rabbit IgG-FITC at 1:200 dilution. Finally, an immunofluorescence assay was carried out according to the protocol by a fluorescence microscope (Olympus, BX51, Japan).

### Measurement of NF-κB p65 activity

The cellular extracts were prepared, the activity of NF-κB p65 transcription factor was measured using NF-κB p50/p65 transcription factor assay kitaccording to the manufacturer’s instructions.

### Western blot analysis

The cells were plated in 6-well plates at density of 1 × 10^6^ per well, TNBS damaged cells were treated with SJZDS (15%) for different times. After the indicated times, cells were collected and lysed in lysis buffer [50 mM Tris (pH 7.4), 150 mM NaCL, 1% Triton X-100, 1% sodium deoxycholate, 0.1% sodium dodecylsulfate (SDS), 1 mmol/l phenylmethylsulphonyl fluoride, and protease inhibitors]. The protein concentration was measured by BCA assay kit, equal amounts of protein were separated by 12.5% SDS-PAGE, then transferred to nitrocellulose membranes. The membranes were blocked and incubated overnight at 4 °C with primary antibodies, followed by incubation for 1 h with the secondary antibodies. Finally, protein expression was detected using the Bio-rad Imaging System (Bio-rad Biosciences, USA).

### Statistical analysis

Results were expressed as the mean ± SEM. Statistical analysis was carried out by the Student’s *t*-test, *P* values less than 0.05 were considered significant. At least three independent experiments were performed.

## Results

### SJZD ameliorated clinical parameters in rats with TNBS-induced colitis

**Fig. 1:**
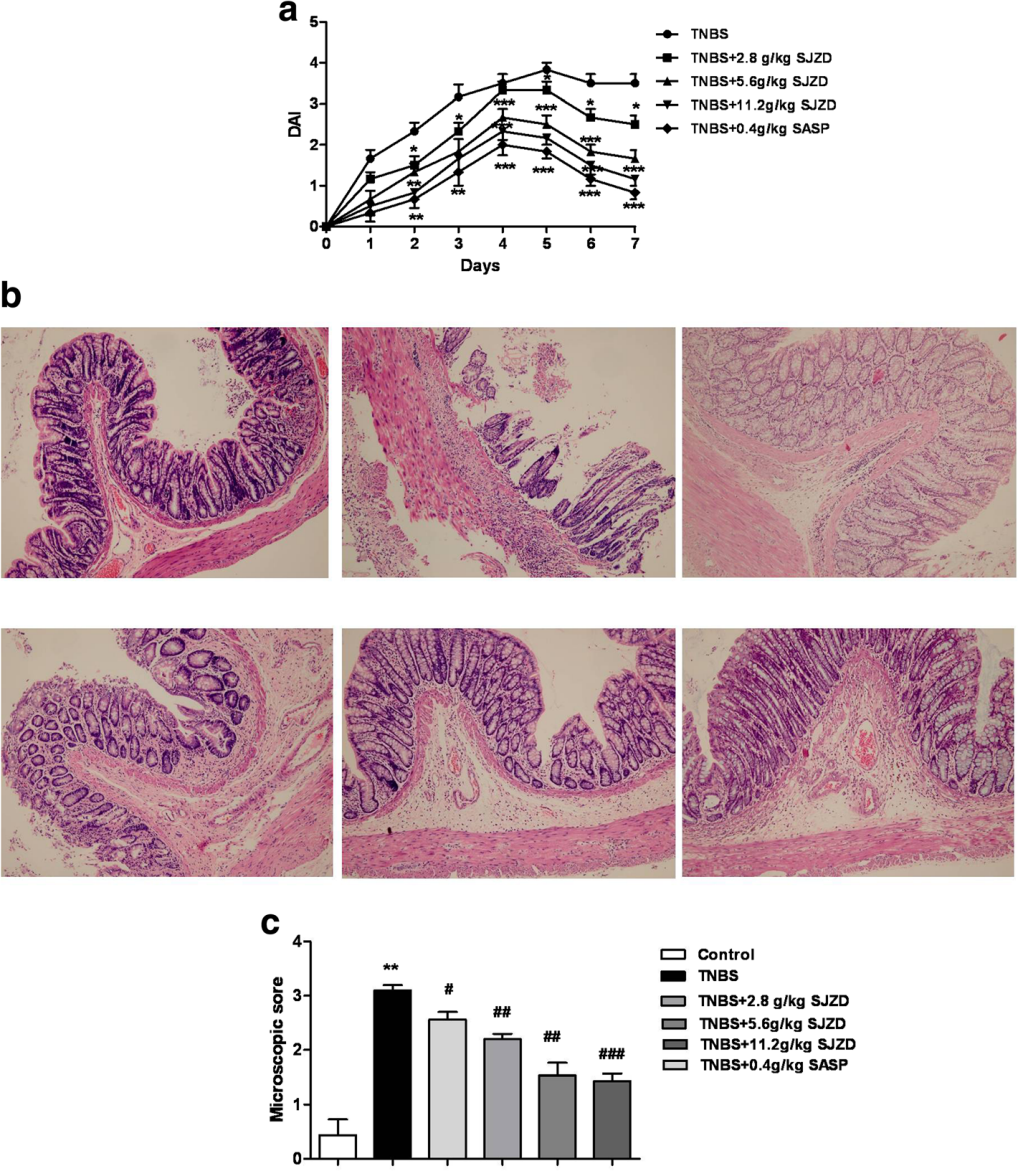
SJZD has a protective effect against TNBS-induced colitis. **a** The disease activity index (DAI) were monitored. **b** Representative histological photograph of colon sections. **c** Microscopic score of sections (*P < 0.05, **P < 0.01 and ***P < 0.001 vs. control group; #*P* < 0.05, ##*P* < 0.01 and ###*P* < 0.001 vs. TNBS-induced colitis group; *n* = 8)

To determine whether oral administration of SJZD could ameliorate the intestinal damage in colitis rats, we induced colitis by administration of TNBS and then treated the rats with SJZD or SASP (positive control) for 7 days. From Fig. 1a, the TNBS group had a sharp increase of the DAI (3.83) from start to day 5, and symptoms were maintained during the experimental period. Compared to the TNBS group, medium and high dose of SJZD significantly decreased the disease severity of TNBS-induced colitis. SASP also reduced the DAImarkedly compared with the TNBS group.

### SJZD decreased histological changes in rats with TNBS-induced colitis

From Fig. 1b and c,histological analysis of the rat colonic tissues revealed a significant reduction in colon inflammation and epithelial cells disruption after the administration of SIZD, compared to the TNBS group. Numerous neutrophils and granulocytes, erosion of mucosal layers were present in the colon of the TNBS group, and a significantly reduced influx of inflammatory cells and intact architecture of the crypts were observed in the colon of the SJZD and SASP treated rats.

### SJZD upregulated the level of claudin-2 in colon of TNBS-induced colitis rats

**Fig. 2:**
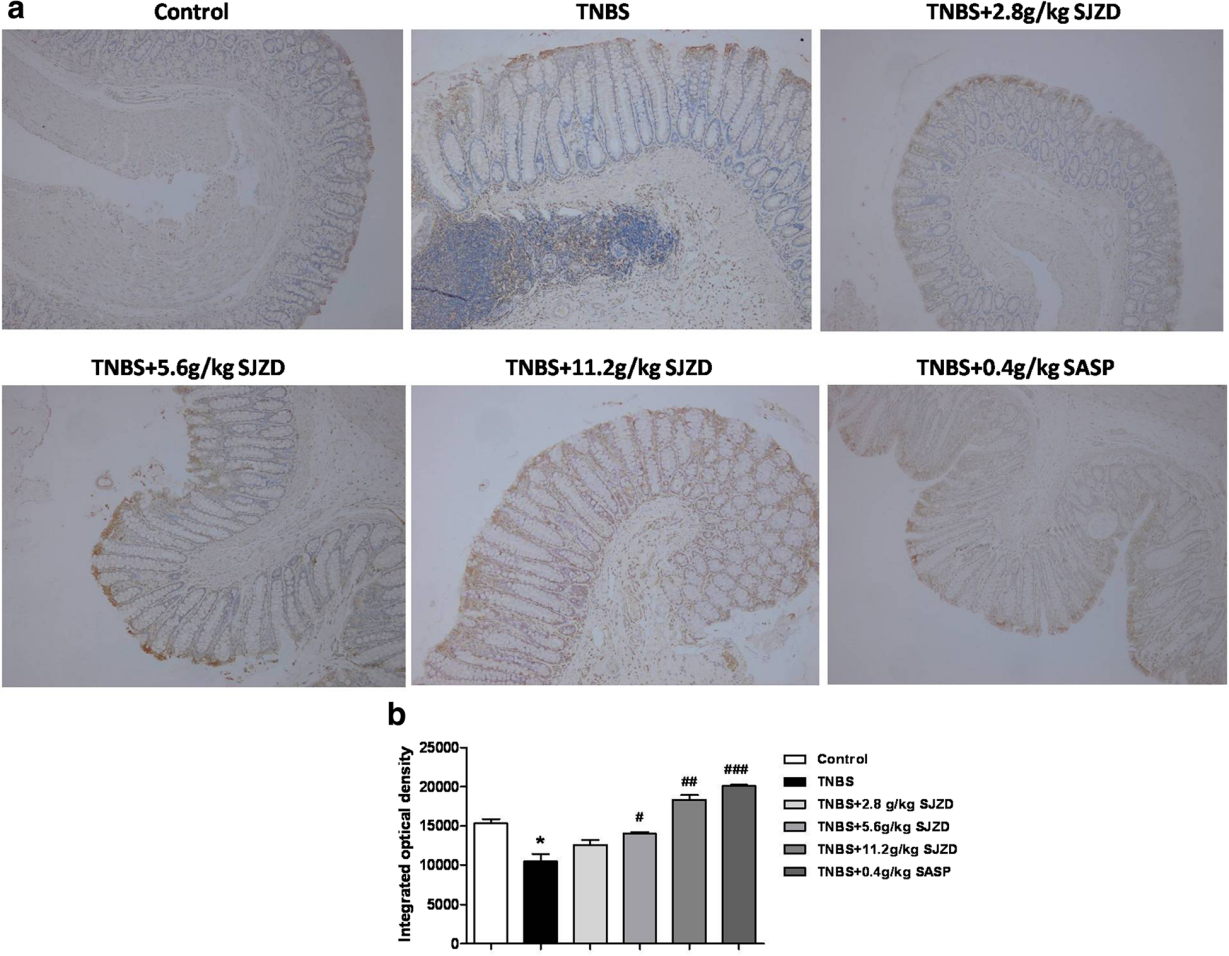
Effect of SJZD on tight junction protein claudin 2 of TNBS-induced colitis in rats by immunochemistry. **a** Representative immunochemical photograph of colon sections, the original magnification was 400×. **b** Quantification of integrated optical density (IOD) in claudin-2. Data represented the mean ± SEM of at least three independent experiments (*P < 0.05 vs. control group; #*P* < 0.05, ##*P* < 0.01 and ###*P* < 0.001 vs. TNBS-induced colitis group; *n* = 8)

Since claudins are the most important transmembrane proteins which can impact the permeability of tight junction, we investigated the expression of claudin-2 in colon tissue by immunochemistry. From Fig. 2, compared to control group, the expression of claudin-2 was downregulated in TNBS-induced colitis rats. The level of claudin-2 was upregulated after the treatment of SJZD and SASP, compared to the TNBS group.

### SJZDS promotes the growth of TNBS-damagedCaco2 cells

**Fig. 3:**
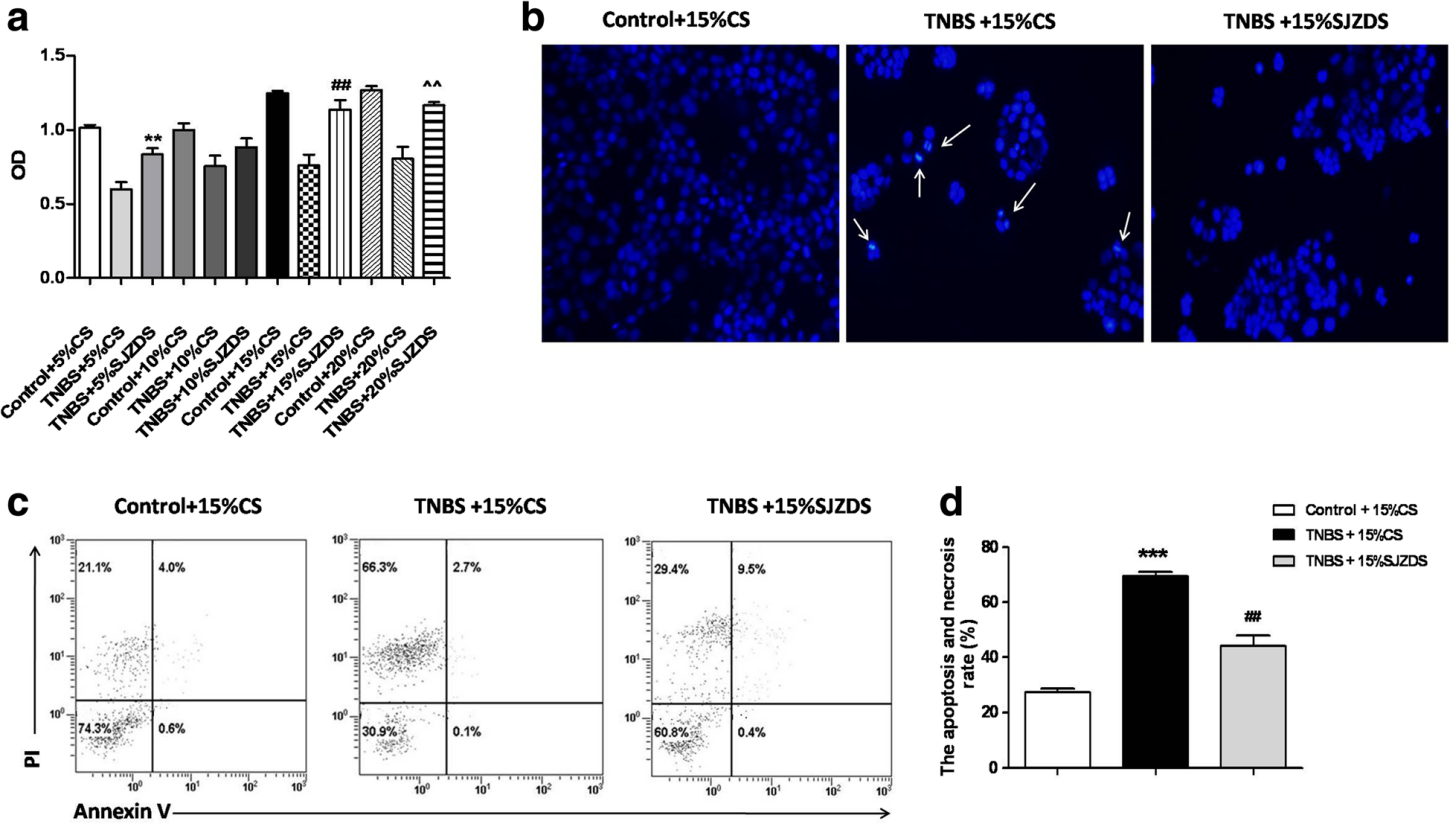
SJZD Serum promoted proliferation and inhibited cell death of TNBS-damaged Caco2 cells. **a** Caco-2 cells were treated with various concentrations of 5–20% Control Serum (CS) or SJZD Serum (SJZDS) for 24 h, the effect of SJZDS on cell viability was measured using the MTT assay. **b** TNBS-damaged Caco2 cells were incubated in the medium with 15% CS or 15% SJZDS for 24 h, then stained with Hoechst 33342 and observed using a fluorescence microscope. Apoptotic cells wereindicated by the arrows (×200magnification). **c** TNBS-damaged Caco2 cells were treated with 15% CS or 15% SJZDS for 24 h. The induction of apoptosis was determined byAnnexin V-FITC/PI staining assay. **d** Quantification of the number of apoptotic cells. Data represented the mean ± SEM of at least three independent experiments (***P < 0.001 vs. control Caco2 cells group; ##*P* < 0.01 vs. TNBS-damaged Caco2 cells group)

The protective effect of SJZDS on TNBS-damagedCaco2 cells was initially determined by the MTT viability assay. From Fig. 3a, it was noted that SJZDSpromoted the proliferation of TNBS-damagedCaco2 cells significantly in a dose-dependent manner, when the concentration of SJZDS achieved15 and 20%, the growth promotion was moresignificant.

### SJZDS inhibited cell apoptosis of TNBS-damaged Caco2 cells

To determine whether the growth protection of SJZDS on TNBS-damaged Caco2 cells was related to the inhibition of cell apoptosis, several apoptotic parameters were measured. Fluorescence microscopic examination indicated that Caco2 cells displayed markednucleus condensation after TNBS treatment, as shown in Fig. 3b. After treatment of 15% SJZDSfor 24 h, the amount of shriveled nucleus decreased. After treatment of TNBS-induced Caco2 cells with 15% SJZDSfor 24 h, the percentage of cell apoptosis decreased from 69.6 ± 1.6% to 44.2 ± 3.8% (Fig. 3c, d).

### SJZDS enhanced the TEER of Caco-2 cells and reduced the permeability

**Fig. 4:**
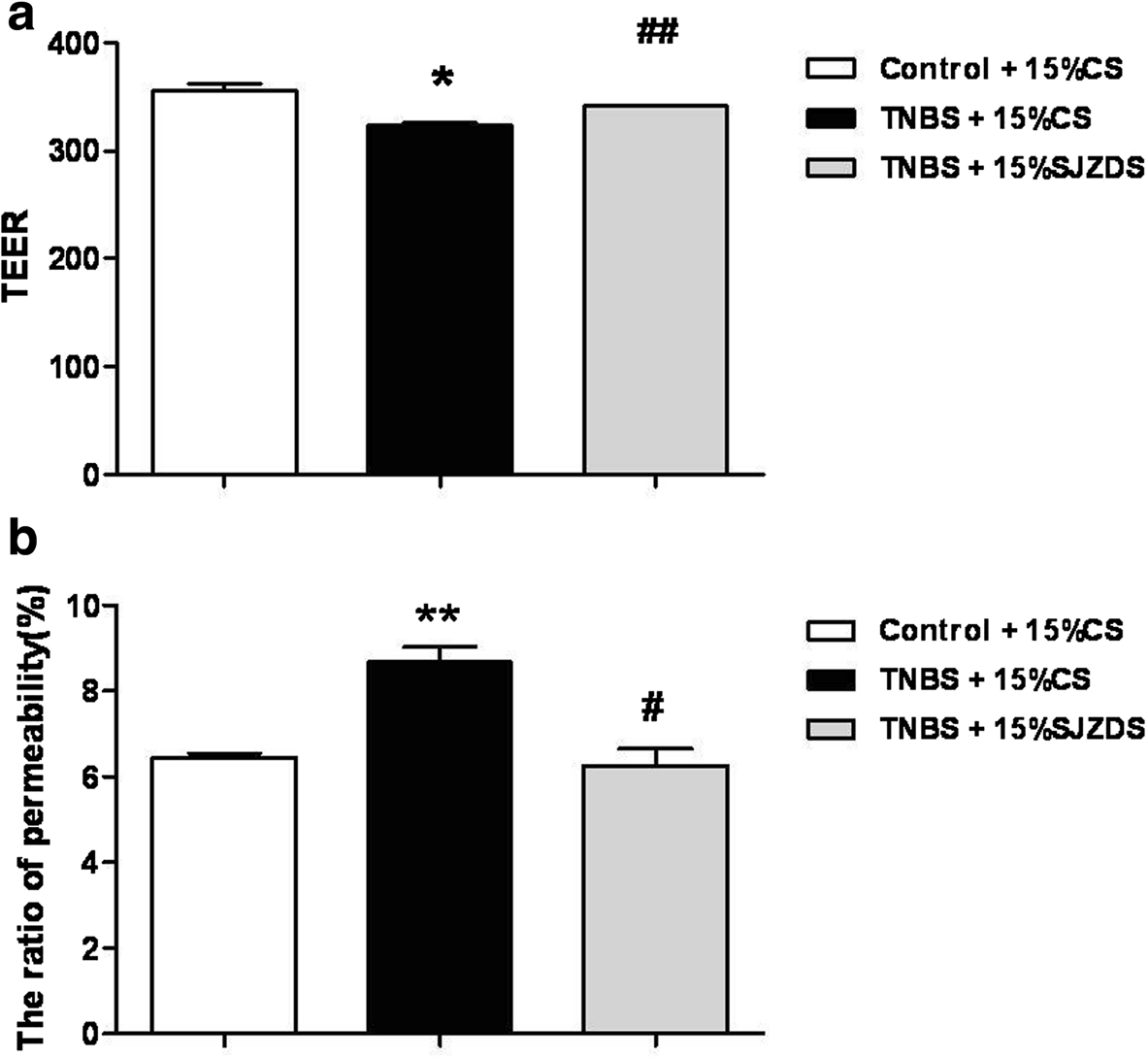
Effect of SJZDS on barrier function of Caco2 cells monolayers. **a** SJZDS significantly increased the TEER of Caco2 cells monolayers. **b** SJZDS markedly attenuated the phenolsulfonphthalein flux across the epithelia monolayers. Data represented the mean ± SEM of at least three independent experiments (*P < 0.05, **P < 0.01 vs. control Caco2 cells group; #*P* < 0.05, ##*P* < 0.01 vs. TNBS-damaged Caco2 cells group)

To investigate whether SJZDS can protect the barrier function of Caco2 cells monolayer, we analyzed the TEER and permeability of Caco2 cells following SJZDS treatment. From Fig. 4a, SJZDSenhanced the TEER of Caco2 cells monolayer at 24 h, as measured by EVOM TEER meter. Besides, SJZDS can reduced the permeabilityof Caco2 cells (Fig. 4b).

### SJZDS protectedbarrier function of Caco2 cells monolayer via regulating claudin-2

**Fig. 5:**
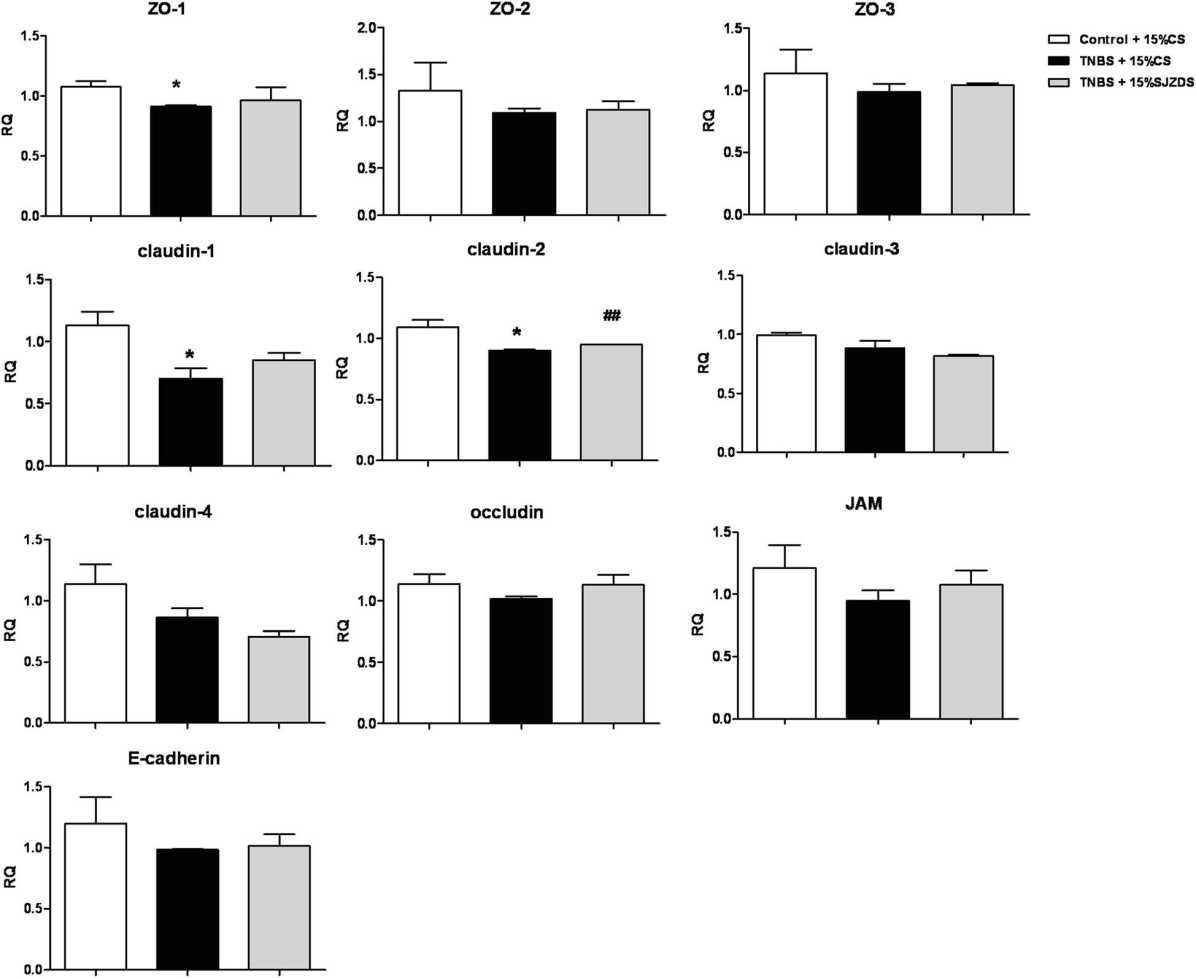
Expression of tight junction proteins in Caco2 cells monolayers. Total RNA was isolated from Caco2 cells, RT-PCR was used to investigated the level of various tight junction proteins. Data represented the mean ± SEM of at least three independent experiments (*P < 0.05 vs. control Caco2 cells group; ##*P* < 0.01 vs. TNBS-damaged Caco2 cells group)

There is increasing evidence that the change of tight junction can influence the intestinal barrier function, which is usually related to an increased permeability and a decrease in TEER [16]. To investigate the underlying protective mechanism of barrier function in Caco2 cells, we analyzed the tight junction protein following SJZDS treatment. From Fig. 5, TNBS can induce the downregulation of different tight junction proteins, and SJZDS promotes the expression of claudin-2, as measured by RT-PCR, but SJZDS has no significant effect on the expression of other tight junction proteins.

**Fig. 6:**
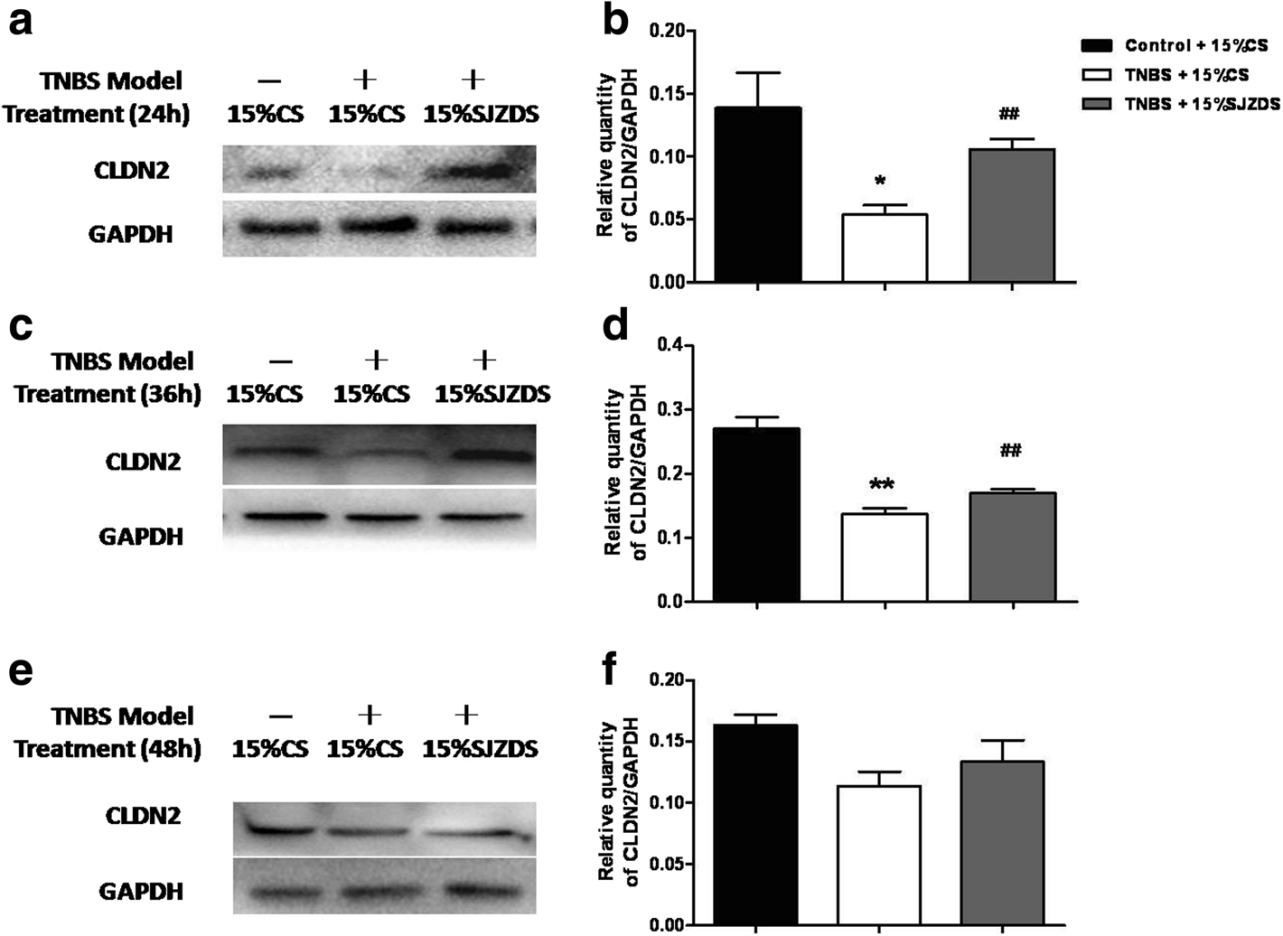
SJZDS modified the expression of claudin 2. **a** The figure showed a representative western blot of claudin 2 in TNBS-damaged Caco2 cells treated by SJZDS for 24 h. **b** Quantification of the amounts of claudin 2relative to GAPDH for 24 h. **c** The figure showed a representative western blot of claudin 2 in TNBS-damaged Caco2 cells treated by SJZDS for 36 h. **d** Quantification of the amounts of claudin 2relative to GAPDH for 36 h. **e** The figure showed a representative western blot of claudin 2 in TNBS-damaged Caco2 cells treated by SJZDS for 48 h. **f** Quantification of the amounts of claudin 2relative to GAPDH for 48 h. Data represented the mean ± SEM of at least three independent experiments (*P < 0.05, **P < 0.01 vs. control Caco2 cells group; ##*P* < 0.01 vs. TNBS-damaged Caco2 cells group)

**Fig. 7:**
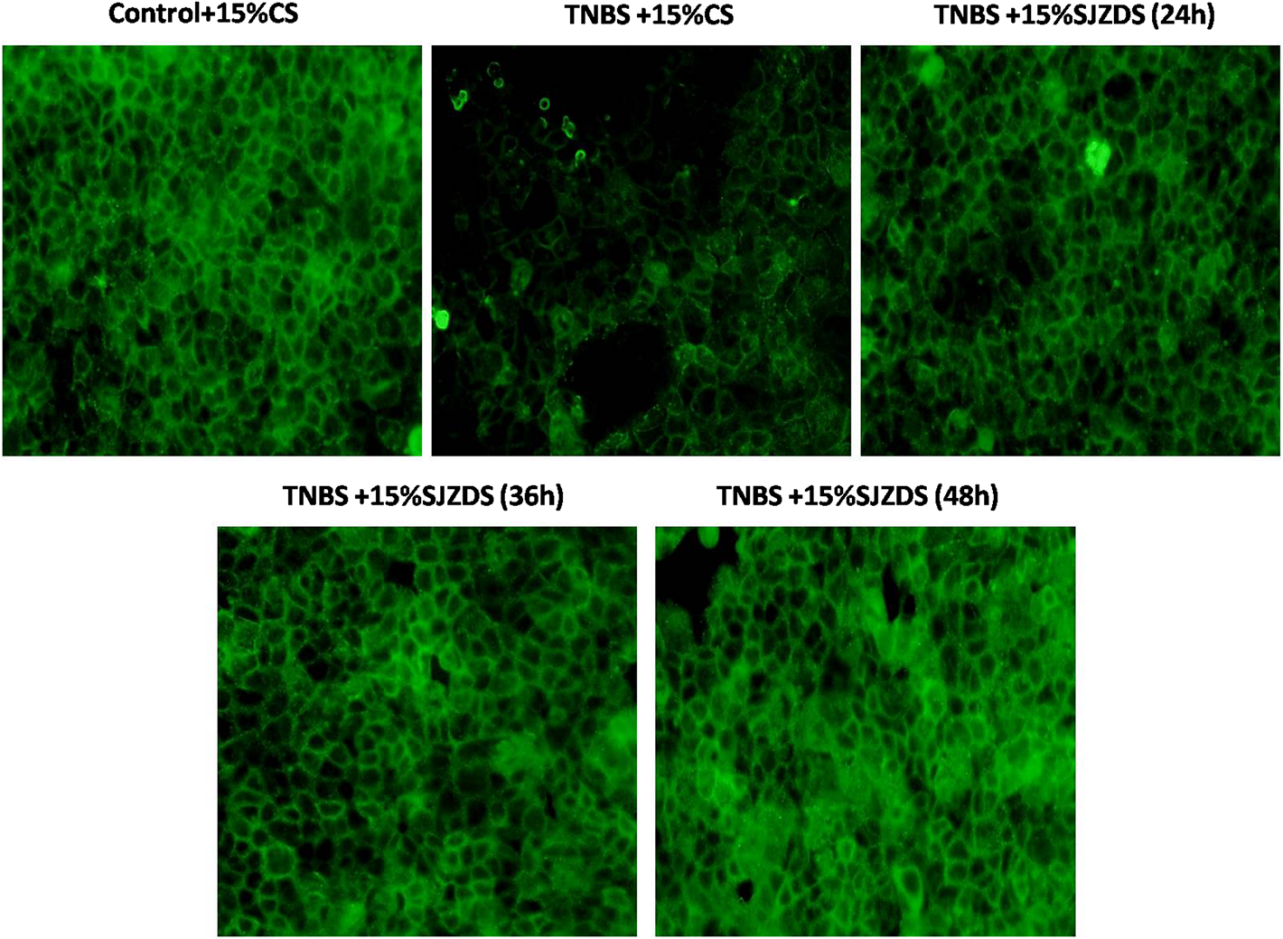
Effect of SJZDS on tight junction protein claudin 2 of TNBS-damaged Caco2 cells by Immunofluorescence. Results were reported from three independent experiments, original magnification was 400×

Furthermore, we quantify the protein expression of claudin-2 by western blot and immunofluorescence. As shown in Fig. 6, the level of claudin-2 was decreased significantly after TNBS damage at 24 h, SJZDS treatment upregulates the expression of claudin-2 for 24 and 36 h, correspondingly, the results of immunofluorescence were similar (Fig. 7).

These results suggested that the protein of claudin-2 might be involved in the protective mechanism of barrier function induced by SJZDS.

### SJZDS upregulated the level of claudin-2 to protect barrier function via inhibiting NF-κB signaling activation

Research reveals that claudin-2 has an important role in inflammation of colon,there is a relationship between claudin-2 and MLCK, which followed the activation of NF-κBpathway [17]. To investigate whether SJZDS has effect on NF-κB signaling in TNBS-damaged Caco2 cells, we measured the activity of NF-κB p65transcription factor and protein expression level of NF-κB p65 and MLCK.

**Fig. 8:**
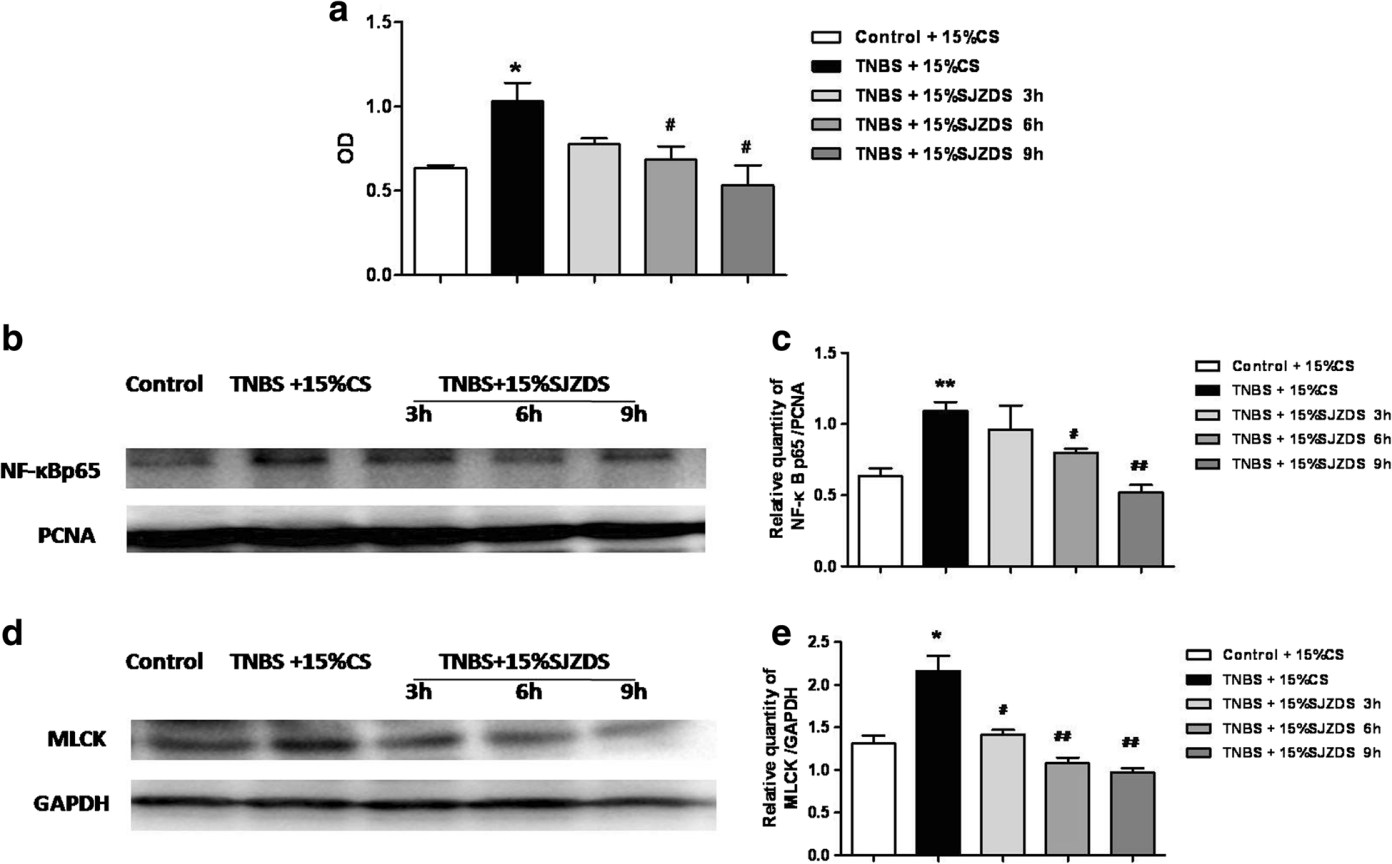
Effect of SJZDS on NF-κB signaling pathway. **a** SJZDS upregulated the activity of NF-κB transcription factor p65. **b** The figure showed a representative western blot of NF-κB transcription factor p65 in TNBS-damaged Caco2 cells treated by SJZDS. **c** Quantification of the amounts of p65relative to PCNA. **d** The figure showed a representative western blot of MLCK in TNBS-damaged Caco2 cells treated by SJZDS. **e** Quantification of the amounts of MLCKrelative to GAPDH. Data represented the mean ± SEM of at least three independent experiments (*P < 0.05, **P < 0.01 vs. control Caco2 cells group; #*P* < 0.05, ##*P* < 0.01 vs. TNBS-damaged Caco2 cells group)

The cellular subfractions were prepared after 15% SJZDS treatment for 3, 6 and 9 h. As shown in Fig. 8a, compared to control group, the activity of NF-κB p65 was increased significantly after TNBS damage for 24 h, after SJZDS treatment for 3 and 6 h, the activity of NF-κB p65 was markedly decreased.

As shown in Fig. 8b and c, the western blot analysis revealed that SJZDS decreased the level of NF-κB p65 protein in TNBS-damaged Caco2 cells after treatment for 6 and 9 h. Moreover, as shown in Fig. 8d and e, treatment with SJZDS resulted downregulation in the level of MLCK at different times.

## Discussion

As an important physical barrier, the intestinal epithelium acts as a guard to protect intestinal tract against bacteria, pathogens and other antigens invaded into the intestinal mucosa and contacted with immune system to prime the abnormal immune responses [18]. The integrity of intestinal barrier can stabilize the entire intestinal ecosystem. And tight junction (TJ) complexes have a decisive effect on the maintenance of barrier integrity [6]. The tight junction (TJ) is a multiprotein complex has roles in selectively regulating paracellular transport of ions and small molecules, preventing endotoxins and microorganisms passing through. Tight junction proteins include transmembrane proteins (such as occludin and claudins), peripheral membraneproteins (such as ZO-1, ZO-2, ZO-3) and regulatory molecules. [19–21]. Defective TJs of the intestinal epithelium have been shown to be an important pathogenic factor of inflammatory bowel diseases [22–25]. Restore the TJ function would be possible as a new target for treatment of IBD.

SijunziDecoction (SJZD) is one of the most famous Traditional Chinese herbal formula and had been used in clinical for treatment of gastrointestinal disorders over 2000 years. It could effectively attenuate nausea,vomitingand diarrhea to restore the homeostasis of the digestive tract in patients [26]. Ten active components in SJZD had been verified, including ginsenoside Rg1, Re, Rb 1, liquiritin, liquiritigenin, glycyrrhizic acid, atractylenolide I, atractylenolide II, atractylenolide III and pachymic acid [14]. Many of these active ingredients had been reported could reduce the symptoms of IBD. For example, GinsenosideRg1, could restrict inflammation progress via improving hypercoagulability and microcirculation, including the prolonged prothrombin time (PT), activated partial thrombin time (APTT) and thrombin time (TT), downregulation of thromboxane B2 (TXB2) to alleviate the symptoms of UC [27]. The butenolidederivatives from Atractylodesmacrocephala showed potent inhibitory effects on NO production in lipopolysaccharide-induced RAW 264.7 cells and exhibited moderate cytotoxicity against HL-60 cell line [28]. Our research group has found Carboxytmethylpachymaran (CMP), extracted from Poriacocos could ameliorate TNF-α-induced damage of intestinal epithelial barrier in Caco-2 monolayers by suppression MLCK-MLC phosphorylationsignaling pathway [29]. However, the research on pharmacological effects and mechanisms of SJZD for treatment IBD is still limited.

Thus, in the present experiments, we explored the effects of SJZD on TNBS-induced colitis rat model, then we investigated TEER, permeability, expression of TJs and NF-κB signaling way of TNBS-damaged Caco2 cells monolayer, further to figure out intestinal barrier protective effect and mechanism of SJZD.

TNBS has long been used as an effective inducer to make mimic experimentally colitis model to investigate the pathophysiological mechanism of inflammatory bowel diseases and to determine the mechanism and efficacy of drugs. TNBS-ethanol administration could induce the inflammation and structure disrupt of mucosa, submucosa and transmural in colon, characterized by Th1-driven inflammation [30], and has been regarded as a similarmodel for CD [31, 32].

In this study, we provided the first evidence that SJZD plays an important role in the protection of TNBS-induced colitis. Our results showed that DAI scores and microscopic scores of colon pathology were significantly reduced in TNBS-induced colitis by SJZD. Moreover, the most important finding was that SJZD could upregulate the level of claudin 2 by immunochemistry in TNBS-induced colitis. These findings suggest that SJZD effectively reduces inflammation and tissue damage of colon in rats.

In the 1980s, Japanese scholars first proposed the serum contained Chinese medicine as an important method of pharmacological study. There are many componentsin Chinese medicine, which may be changed in the period of absorption and metabolism in vivo [33, 34]. The componentsafter absorption and metabolism, would be transported to the target organ or tissue to make effect [35]. Therefore, we can use serum of SJZD (SJZDS) to further explore the effect and mechanism of SJZD in vitro*.*

Human Caco2 intestinal epithelial cells are polarized epithelial cells which could form apical junction complexes, resulting in high electrical resistance, are useful for studying effects of therapies on permeability.

Then, we investigated the effect and mechanism of SJZDS on intestinal barrier protection in TNBS damaged Caco2 cells. Our results showed TNBS could inhibit proliferation and induce apoptosis of Caco2 cells, lead morphological changes by Hoechst 33342. SJZDS was able to antagonize this effect, which could promote proliferation and reduce apoptosis of cells.

Restoring physical barrier function is crucial in the treatment of intestinal inflammatory disorders. After 24 h TNBS damage on Caco2 cell monolayers, TEER decreased from 355.2 ± 6.9 to 324 ± 1.8 Ω cm^2^, then in SJZDS-treated Caco2 cells, TEER increased from 324 ± 1.8 to 341 ± 0.88 Ω cm^2^. TNBS induced drop in Caco2 TEER correlated directly with the increase in paracellular permeability. SJZDS could decrease paracellular permeability of Caco2 cell monolayers for 24 h.

To explain the relationship between the TJ proteins and barrier function, we analyzed a series of TJ proteins by RT-PCR, the results showed TNBS could downregulate the mRNA level of ZO-1, claudin-1, claudin-2 in Caco2 cells, after SJZDS treatment, the mRNA level of claudin-2 was upregulated. So we next focused the target of SJZDS on claudin-2, the treatment of TNBS-damaged Caco2 cells with SJZDS resulted in the increase in the expression of claudin-2 by western blot and immunofluorence.

NF- κB, an important nuclear transcriptional factor, its signaling pathway has been involved in numerous physiological processes, including regulation of inflammation response and innate immunity, and therefore may be participation in IBD in multiple ways [36]. The increased expression of NF-κB in the acute phase of colitis had been proved and is consistent with its role in the onset of experimental colitis [37]. SJZDS could significantly inhibit NF- κB p65 activity and expression in nuclear after treatment for 3 h and 6 h, make it impossible to block downstream cytokine and inflammatory factors expression.

TJs are closely link to various intracellular signaling molecules and are regulated by multiple signal transduction pathways. Myosin light chain kinase (MLCK) is classically known to be required for the contraction of actomyosin via the phosphorylation of myosin light chain (MLC) [38] and has also been reported to be expressed in the human intestinal tissue with IBD [39]. It is also essential to regulation the permeability of epithelial barrier and affects production of inflammatory cytokine, such as TNFα, in the inflamed intestinal tissues. Inhibition of MLCK can attenuate TNFα induced nuclear translocation of p65 and phosphorylation of IκB, which indicates MLCK play a role in inducing activation of NF-κB [40]. We next investigated the level of MLCK. The results showed that the expression of MLCK was increased in TNBS-damaged Caco2 cells, SJZDS decreased the level of MLCK at different times.

## Conclusions

In this study, our results demonstrate that SJZD ameliorates the severity of TNBS-induced colitis and downregulates the level of claudin-2 in colonic tissues. In vitro data showsSJZDS promotes proliferation and inhibits apoptosis ofTNBS-damaged Caco2 cells. In Caco2 cell monolayers, SJZDS increases theTEER andreduces permeability after TNBS damage, we finds that the barrier protective effect of SJZDS is mediated through claudin-2 and NF-κB pathway, including upregulation of claudin-2, decreased activity of NF-κB p65, reduced expression of NF-κB p65 and MLCK. Taken together, these findings provide evidence that, SJZD, is a potential protective agent ofintestinal barrier function. Further investigation into the mechanism of SJZD on intestinal barrier as well as in vivo research is required.

## Abbreviations

CD: Crohn’s disease
H&E: Hematoxylin and eosin
IBD: Inflammatory bowel disease
SASP: Salazosulfapyridine
SJZD: SijunziDecoction
SJZDS: SijunziDecoction serum
TEER: Transepithelial electrical resistance
TJ: Tight junction.
TNBS: 6-trinitrobenzene sulfonic acid
UC: Ulcerative colitis
ZO1: Zonulaoccludens 1

## References

[1] Podolsky DK (1991). Inflammatory bowel disease (1). N Engl J Med.

[2] Yamamoto-Furusho JK, Podolsky DK (1991). Innate immunity in inflammatory bowel disease. N Engl J Med.

[3] Fiocchi C (1998). Inflammatory bowel disease: etiology and pathogenesis. World J Gastroenterol.

[4] Podolsky DK (2002). Inflammatory bowel disease. N Engl J Med.

[5] Bouma G, Strober W (2003). The immunological and genetic basis of inflammatory bowel disease. Nat Rev Immunol.

[6] Mitic LL, Van Itallie CM, Anderson JM (2000). Molecular physiology and pathophysiology of tight junctions I. Tight junction structure and function: lessons from mutant animals and proteins. Am J Physiol Gastrointest Liver Physiol.

[7] Turner JR (2009). Intestinal mucosal barrier function in health and disease. Nat Rev Immunol.

[8] Pithadia AB, Jain S (2011). Treatment of inflammatory bowel disease (IBD). Pharmacol Rep.

[9] Triantafyllidi A, Xanthos T, Papalois A, Triantafillidis JK. Herbal and plant therapy in patients with inflammatory bowel disease. Ann Gastroenterol. 2015;28(2):210–20. PMC4367210 25830661

[10] Wang X, Fan F, Cao Q (2016). Modified Pulsatilla decoction attenuates oxazolone-induced colitis in mice through suppression of inflammation and epithelial barrier disruption. Mol Med Rep.

[11] Xu BL, Zhang GJ, Ji YB. Active components alignment of Gegenqinlian, decoction protects ulcerative colitis by attenuating inflammatory and oxidative stress. J Ethnopharmacol. 2015;13(162):253–60. 10.1016/j.jep.2014.12.042 25557032

[12] Fan H, Liu XX, Zhang LJ, Hu H, Tang Q, Duan XY, Zhong M, Shou ZX (2014). Intervention effects of QRZSLXF, a Chinese medicinal herb recipe, on the DOR-β-arrestin1-Bcl2 signal transduction pathway in a rat model of ulcerative colitis. J Ethnopharmacol.

[13] Ji YF, Wang RJ, Li XB (2016). Research progress on chemical constituents and pharmacological effects of Sijunzi decoction. ChinTrad Herb Dr.

[14] An K, Jin-Rui G, Zhen Z, Xiao-Long W. Simultaneous Quantification of Ten Active Components in Traditional Chinese Formula Sijunzi Decoction Using a UPLC-PDA Method. J Anal Methods Chem. 2014;2014(11):570359. 10.1155/2014/570359 PMC4054979 24963442

[15] Zhao SY, Wang CJ (2014). Influence of Sijunzi decoction’s on T lymphocyte subsets of gastric mucosa in rats with spleen-deficiency syndrome. Chin J Integr Trad Med Dig.

[16] Klingberg TD, Pedersen MH, Cencic A, Budde BB (2005). Application of measurements of transepithelial electrical resistance of intestinal epithelial cell monolayers to evaluate probiotic activity. Appl Environ Microbiol.

[17] Nishida M, Yoshida M, Nishiumi S, Furuse M, Azuma T (2013). Claudin-2 regulates colorectal inflammation via myosin light chain kinase-dependent signaling. Dig Dis Sci.

[18] Sierro F, Dubois B, Coste A, Kaiserlian D, Kraehenbuhl JP, Sirard JC (2001). Flagellin stimulation of intestinal epithelial cells triggers CCL20-mediated migration of dendritic cells. Proc Natl Acad Sci USA.

[19] Garrote JA, Gómez-González E, Bernardo D, Arranz E, Chirdo F (2008). Celiac disease pathogenesis: the proinflammatory cytokine network. J Pediatr Gastroenterol Nutr.

[20] Ivanov AI, Parkos CA, Asma N (2010). Cytoskeletal regulation of epithelial barrier function during inflammation. Am J Pathol.

[21] Hartsock A, Nelson WJ (2008). Adherens and tight junctions: structure, function and connections to the actin cytoskeleton. Biochimica EtBiophysica Acta Biomembranes.

[22] Hermiston ML, Gordon JI (1995). Inflammatory bowel disease and adenomas in mice expressing a dominant negative N-cadherin. Science.

[23] Costantini TW, Peterson CY, Kroll L, Loomis WH, Eliceiri BP, Baird A, Bansal V (2009). Coimbra RRole of p38 MAPK in burn-induced intestinal barrier breakdown. J Surg Res.

[24] Yasuda T, Takeyama Y, Ueda T, Shinzeki M, Sawa H, Nakajima T, Kuroda Y (2006). Breakdown of intestinal mucosa via accelerated apoptosis increases intestinal permeability in experimental severe acute pancreatitis. J Surg Res.

[25] Seo GS, Jiang WY, Park PH, Sohn DH, Cheon JH, Lee SH (2014). Hirsutenone reduces deterioration of tight junction proteins through EGFR/Akt and ERK1/2 pathway both converging to HO-1 induction. Biochem Pharmacol.

[26] Yang L, Yang J, Cai Z (2006). Chemical investigation on Sijunzi decoction and its two major herbs Panax ginseng, and Glycyrrhizauralensis, by LC/MS/MS. J Pharm Biomed Anal.

[27] Hao WW, Wen HZ, Ma GT, Tang ZP, He XY, Li J, Li NN, Liu YT (2013). Ginsenoside-Rg1regulates blood coagulation in DSS-induced colitis mice. Chin J Integr Trad Med Dig.

[28] Guo F, Li Z, Xu X, Wang K, Shao M, Zhao F, Wang H, Hua H, Pei Y, Bai J (2016). Butenolide derivatives from the plant endophytic fungus Aspergillusterreus. Fitoterapia.

[29] Zhang J, Lu Y, Wei J, Li L, Han L (2016). Protective effect of carboxytmethylpachymaran on TNF-α-induced damage in Caco-2 cell monolayers. Int J BiolMacromol.

[30] Neurath M, Fuss I, Strober W (2000). TNBS-colitis. Int Rev Immunol.

[31] Jurjus AR, Khoury NN, Reimund JM (2004). Animal models of inflammatory bowel disease.J PharmacolToxicol. Methods.

[32] Brenna Ø, Furnes MW, Drozdov I, van BeelenGranlund A, Flatberg A, Sandvik AK, Zwiggelaar RT, Mårvik R, Nordrum IS, Kidd M, Gustafsson BI (2013). Relevance of TNBS-colitis in rats: a methodological study with endoscopic, Histologic and transcriptomic [corrected] characterization and correlation to IBD. PLoS One.

[33] Dou ZH, Luo L, Chen JY, AN LP, YANG AH (2013). Analysis of lignans in serum containing drug of Schisandrachinensis by UPLC-MS/MS. Chin J Clin Pharmacol.

[34] Wang X, Sun W, Sun H, Lv H, Wu Z, Wang P, Liu L, Cao H (2008). Analysis of the constituents in the rat plasma after oral administration of Yin Chen Hao Tang by UPLC/Q-TOF-MS/MS. J Pharm Biomed Anal.

[35] Wang P, Liang Y, Zhou N, Chen B, Yi L, Yu Y, Yi Z (2007). Screening and analysis of the multiple absorbed bioactive components and metabolites of dangguibuxue decoction by the metabolic fingerprinting technique and liquid chromatography/diode-array detection mass spectrometry. Rapid Commun Mass Spectrum.

[36] Anselmi L, Huynh J, Duraffourd C, Jaramillo I, Vegezzi G, Saccani F, Boschetti E, Brecha NC, De Giorgio R, Sternini C (2015). Activation of μ opioid receptors modulates inflammation in acute experimental colitis. Neurogastroenterol Motil.

[37] Funakoshi T, Yamashita K, Ichikawa N, Fukai M, Suzuki T, Goto R, Oura T, Kobayashi N, Katsurada T, Ichihara S, Ozaki M, Umezawa K, Todo S (2012). A novel NF-κB inhibitor, dehydroxymethylepoxyquinomicin, ameliorates inflammatory colonic injury in mice. J Crohns Colitis.

[38] Liu X, Xu J, Mei Q, Han L, Huang J (2013). Myosin light chain kinase inhibitor inhibits dextran sulfate sodium-induced colitis in mice. Dig Dis Sci.

[39] Blair SA, Kane SV, Clayburgh DR, Turner JR (2006). Epithelial myosin light chain kinase expression and activity are upregulated in inflammatory bowel disease. Lab Invest.

[40] Wadgaonkar R, Linz-McGillem L, Zaiman AL, Garcia JG (2005). Endothelial cell myosin light chain kinase (MLCK) regulates TNFalpha-induced NFkappaB activity. J Cell Biochem.

